# Computational tools to facilitate in situ structure determination using cryo-ET

**DOI:** 10.1063/4.0001018

**Published:** 2025-10-27

**Authors:** Alberto Bartesaghi

**Affiliations:** Duke University, Durham, NC, USA

## Abstract

Cryo-electron tomography combined with sub-volume averaging is a powerful technique capable of determining the structure of proteins imaged within the native context of cells at near-atomic resolution. While high-throughput techniques for sample preparation and tilt-series acquisition are beginning to provide sufficient data to allow structural studies of proteins at physiological concentrations, the complex data analysis pipeline and the demanding storage and computational requirements pose major barriers for the development and broader adoption of this technology. Here, I will present recent advances in image processing that have facilitated the conversion of raw tilt series into high- resolution structures by streamlining the analysis of large datasets containing hundreds of tilt series. We validated these tools on challenging datasets, demonstrating their effectiveness at uncovering cellular patterns and revealing conformational heterogeneity in situ. Availability of these tools will contribute to broadening the adoption of cryo-ET as a powerful technique for studying proteins in their native environment at high-resolution.

